# *Trichoderma carraovejensis*: a new species from vineyard ecosystem with biocontrol abilities against grapevine trunk disease pathogens and ecological adaptation

**DOI:** 10.3389/fpls.2024.1388841

**Published:** 2024-05-21

**Authors:** Laura Zanfaño, Guzmán Carro-Huerga, Álvaro Rodríguez-González, Sara Mayo-Prieto, Rosa E. Cardoza, Santiago Gutiérrez, Pedro A. Casquero

**Affiliations:** ^1^ Research Group of Engineering and Sustainable Agriculture, Natural Resources Institute, Universidad de León, León, Spain; ^2^ Area of Microbiology, University School of Agricultural Engineers, Universidad de León, Ponferrada, Spain

**Keywords:** *Trichoderma carraovejensis*, phylogeny, biological control, grapevine trunk diseases, ecology

## Abstract

*Trichoderma* strains used in vineyards for the control of grapevine trunk diseases (GTDs) present a promising alternative to chemical products. Therefore, the isolation and characterization of new indigenous *Trichoderma* strains for these purposes is a valuable strategy to favor the adaptation of these strains to the environment, thus improving their efficacy in the field. In this research, a new *Trichoderma* species, *Trichoderma carraovejensis*, isolated from vineyards in Ribera de Duero (Spain) area, has been identified and phylogenetically analyzed using 20 housekeeping genes isolated from the genome of 24 *Trichoderma* species. A morphological description and comparison of the new species has also been carried out. In order to corroborate the potential of *T. carraovejensis* as a biological control agent (BCA), confrontation tests against pathogenic fungi, causing various GTDs, have been performed in the laboratory. The compatibility of *T. carraovejensis* with different pesticides and biostimulants has also been assessed. This new *Trichoderma* species demonstrates the ability to control pathogens such as *Diplodia seriata*, as well as high compatibility with powdered sulfur-based pesticides. In conclusion, the autochthonous species *T. carraovejensis* can be an effective alternative to complement the currently used strategies for the control of wood diseases in its region of origin.

## 1 Introduction

*Trichoderma* is a fungal genus that belongs to one of the largest classes of *Sordariomycetes* (phylum *Ascomycota*). Inside this class, the largest number of known genera is found within the order *Hypocreales*, which comprises half of the sequenced genomes in *Sordariomycetes* species. In this order, there are approximately 400 different species of the genus *Trichoderma* that are included. These data have been generated thanks to the evolution of molecular methods (Cai and Druzhinina, 2021). Most species of *Trichoderma* could be clustered into three big groups—clade *Harzianum/Virens*, section *Trichoderma*, and section *Longibrachiatum*—according to phylogenetic studies (Kubicek et al., 2019). *Harzianum* is one of the most important clades of species of this genus with application in biological control since most of the species applied in agriculture (Woo et al., 2014; Tyśkiewicz et al., 2022) have been putatively identified as *Trichoderma harzianum* (Proietti et al., 2018; Barrera et al., 2021). The classification of *Trichoderma* species belonging to the *Harzianum* clade represents a great challenge for researchers since several issues make this task difficult to manage, e.g., the problem generated by its sexual stage or teleomorph, named *Hypocrea*; the identification of gamospecies, cryptic species; and most especially, the use of ITS for identification (Druzhinina et al., 2010). The general use of ITS regions (White et al., 1990) (ITS1 or ITS2) revealed that closely related *Trichoderma* species have the same ITS phylotypes, especially for infrageneric groups such as the *Harzianum* clade.

Recently, accurate and advisable new forms to identify a species were used to identify new *Trichoderma* isolates according to the International Commission on *Trichoderma* Taxonomy (ICTT, https://trichoderma.info/2021/04/26/molecular-identification-protocol-for-trichoderma/). Moreover, the species *T. harzianum* is the most common species (*T. harzianum sensu stricto*) in the *Harzianum* clade, but an inaccurate identification could lead to mistakes. A recent review showed that many *Trichoderma* species were not well identified or misclassified in the NCBI GenBank (Cai and Druzhinina, 2021). For example, *T. harzianum* T22 was re-identified as *Trichoderma afroharzianum* T22 and new species appeared in this clade such as *Trichoderma lentiforme* and *T. lixii* (Chaverri et al., 2015). Nowadays, new genetic tools such as next-generation sequencing (NGS) techniques and the standardized protocol of ICTT allow us to identify more accurately any species.

A great number of *Trichoderma* species have been shown to act as BCAs, due to their antagonistic ability against other pathogenic microorganisms (Rees et al., 2020; Mukherjee et al., 2022). The mechanisms of action of *Trichoderma* species acting as BCAs are very diverse, such as being able to produce extracellular hydrolytic enzymes (CAZymes) such as glucanases, chitinases, and proteases; degrade polymers of the cell wall and membrane of phytopathogens; secrete antimicrobial compounds (antibiosis); and compete for a niche (nutrients, tissues, etc.) (Zeilinger et al., 2016; Woo et al., 2022). These fungi also have the ability to stimulate plant growth and defense responses (Druzhinina et al., 2011; Hermosa et al., 2014). *Trichoderma* species are widely distributed, and they can be found in very diverse ecosystems, such as decaying wood, soil, bark, leaves, or the root system of plants, as well as endophytes in plant tissues (Kredics et al., 2021). Currently, *Trichoderma* is being introduced in viticulture as a promising preventative method in combination with other sustainable solutions against grapevine trunk diseases (GTDs) (Mondello et al., 2018). Different studies have demonstrated its effectiveness against some of the most common grapevine trunk diseases, such as esca disease (Bigot et al., 2020; Carro-Huerga et al., 2020), black-foot disease (Berlanas et al., 2018; van Jaarsveld et al., 2021), or *Botryosphaeria* dieback (Silva-Valderrama et al., 2021; Pollard-Flamand et al., 2022), among others. Some *Trichoderma* isolates have demonstrated the ability to generate induced resistance in grapevine plants against the pathogen *Erysiphe necator*, which causes powdery mildew (Sawant et al., 2020). It was also demonstrated that *Trichoderma* was able to mycoparasite *E. necator* (Sawant et al., 2017). One of the most studied major commercialized biocontrol agents in viticulture is *Trichoderma atroviride* SC1, which has been described as an effective biocontrol agent against pathogens that cause grapevine trunk diseases (Berbegal et al., 2020; Lazazzara et al., 2021; Leal et al., 2023). However, some studies have shown that these strains that are commercially used are not always working, so the search for new more effective *Trichoderma* species is needed (Martínez-Diz et al., 2021).

GTDs are causing severe damage in vineyards around the world, with their incidence increasing in recent years. In Castilla-La Mancha, Spain, the main wine-growing area in extension, symptoms of GTDs were shown in 38.3% of the varieties evaluated (Chacón-Vozmediano et al., 2021). In Navarra, the incidence of GTDs in young tempranillo plants on different rootstocks was evaluated. The evaluation data for GTDs for the 2016, 2017, and 2018 seasons showed, in the case of eutypiosis, a cumulative incidence of over 10% for rootstock 161-49 C (Gramaje et al., 2020).

In recent years, the use of effective chemicals against GTDs has been reduced due to the increasing restrictions related to the high toxicity of these products for health and the environment (Decoin, 2001), which has led to the search for solutions based on BCAs. Currently, commercial *Trichoderma*-based products account for 21% of registered products in the European Union (EU Pesticides Database, 2024) and 2.5% in the United States (Biopesticide Active Ingredients | US EPA, 2024). Therefore, *Trichoderma* represents a present and future alternative for agriculture through the use of different mechanisms of action against the main grapevine diseases.

One of the main strategies to be employed could be the use of indigenous organisms for the biocontrol of vineyard areas affected by GTDs. Some studies (Álvarez-Pérez et al., 2017; Carro-Huerga et al., 2021) have shown a better adaptation of these BCAs to the environmental conditions and a greater capacity of protection and defense of the vine plants. So, this could be an alternative to pesticides and the use of microorganisms adapted to viticulture soil and management.

The aims of this study were to describe a new autochthonous *Trichoderma* species, strain T154, named after this study *T. carraovejensis*, which has been isolated from vineyards in Castilla y León region (Spain), and to analyze its potential as a BCA against GTDs, as well as its compatibility with other biostimulants and fungicides.

## 2 Materials and methods

### 2.1 Fungal strains

*Trichoderma* sp. strain T154 was isolated from the wood of *Vitis vinifera* cv. Tempranillo, at Winery “Pago de Carraovejas” in Peñafiel, 41°35′51*"*N, 4°07′22*"*W (Valladolid, Spain) (Carro-Huerga et al., 2020). This strain was stored at the “Laboratorio de Diagnóstico de Plagas y Enfermedades Vegetales” (Plant and Pest Diagnostic Laboratory) (LDPEV) Universidad de León, Spain, under accession code ULET154.

The four *Trichoderma* strains used for morphological comparison purposes were as follows: *T. lentiforme* CBS 100542, *Trichoderma atrobrunneum* CECT 20730, *Trichoderma guizhouense* CECT 20731, and *Trichoderma harzianum sensu stricto*, which have been deposited at LDPEV under accession number ULET87 (University of Leoín, Spain). This strain (ULET87) was isolated from vineyard soils in Castilla y León region, and it has been assayed against *Phaeoacremonium minimum* during an *in-vitro* test with a significant percentage of biocontrol (Carro-Huerga et al., 2023).

Pathogens used in biocontrol assays, available at the LDPEV collection, belong to the main GTDs and most aggressive pathogens described in the literature (Martín and Cobos, 2007) in our region: *Diplodia seriata* (ULEP32), which is the most representative and aggressive pathogen of *Botryosphaeria* dieback disease, was isolated from plants with severe symptoms of xylem necrosis and shoot dieback in Castilla y León (Spain). Two of the main pathogens that cause Petri and esca diseases were also isolated: *P. minimum* Y038-05-3, a very aggressive isolate, was isolated from vine plants in Valle Benavente (Spain) (Martín and Martín, 2013) (stored at LDPEV under the accession number ULEA16); and finally, *Phaeomoniella chlamydospora* isolate Y-116-18-03c, another causal agent described as an important pathogen that causes symptoms of esca disease (Martín et al., 2012) (stored at LDPEV under the accession number ULEC21), was isolated.

### 2.2 Isolation of fungal strains

The T154 isolate was isolated in a previous study (Carro-Huerga et al., 2020), carried out in the Pago de Carraovejas winery (Peñafiel, Spain). Briefly, vine bark samples were taken from various plants using pruning shears, which were disinfected with 70% ethanol between samples. These wood pieces were preserved in clean plastic bags with hermetic seals at 4°C. The bark samples were then disinfected in a 1.5% sodium hypochlorite solution for 1 min and then washed with plenty of sterile distilled water. These bark fragments were dried out for 15 min in a laminar flow chamber and cut with a sterile scalpel. Subsequently, seven wood chips (approx. 1–2 mm in diameter; approx. 0.5–1 cm in length) per plate were placed on Rose Bengal-Chloramphenicol Agar plates (Conda Laboratory, Madrid, Spain). These cultures were incubated at 25°C. After 3–5 days, mycelial growth was observed on each of the tissue pieces of wood, and those that were morphologically identified with *Trichoderma* were isolated and cultured on PDA plates (Sigma-Aldrich Chemie GmbH, Steinheim, Germany). The morphology of the *Trichoderma* strain was evaluated according to Harman and Kubicek (1998a).

### 2.3 *Trichoderma* identification

For a preliminary identification of the *Trichoderma* isolates recovered in the present study, a strategy based on PCR amplification, nucleotide sequencing of ITS regions, and Blastn comparison to sequences in the non-redundant GenBank NCBI database (http://www.ncbi.nlm-nih.gov), using the BLASTn program (http://www.ncibi.nlm.nigh.gov/BLAST), was followed as described previously (Carro-Huerga et al., 2020).

### 2.4 Genome sequencing

The genome sequence from the T154 isolate was generated by Macrogen Inc. (Seoul, Korea; https://dna.macrogen.com) using an Illumina platform. Sequence assemblies were generated using Platanus Allee (v2.2.2) software (Kajitani et al., 2019). This Whole Genome Shotgun project has been deposited at DDBJ/ENA/GenBank under the accession JAZAQE000000000. The version described in this paper is version JAZAQE010000000.

### 2.5 Phylogenetic analyses

Nucleotide sequences from 20 *Trichoderma* housekeeping (=HK) genes (**Supplementary Table S1**) retrieved from the genome of 24 *Trichoderma* species (Gutiérrez et al., 2021) were used to infer a *Trichoderma* species tree.

The sequences of each gene from all the *Trichoderma* spp. analyzed were individually aligned by MUSCLE software as implemented in MEGA X (Kumar et al., 2018), and then the alignments were concatenated using the Sequence Matrix software (Vaidya et al., 2011). The resulting concatenated alignment was then subjected to maximum likelihood (ML) analysis as implemented in the program IQ-TREE version 1.6.12 (Nguyen et al., 2014). A second concatenated-partitioned tree was constructed by selecting for each gene the best-fit evolutionary nucleotide model deduced from the previous IQ-TREE analysis. Finally, both concatenated (non-partitioned and partitioned) alignments were subjected to ML analysis as implemented in IQ-TREE. Branch support was determined by bootstrap analysis using 1,000 pseudoreplicates. In addition, to assess the consistency of trees inferred from the 20 housekeeping genes, a gene concordance factor (GCF) analysis was performed as described by Minh et al. (2020) and Gutiérrez et al. (2021).

In order to support these studies, three other trees were inferred using partial amino acid sequences deduced from coding sequences of three housekeeping genes [*acl1* (ATP citrate lyase), *rpb2* (RNA polymerase 2nd largest subunit), and *tef1* (translation elongation factor 1-alpha)], which were retrieved from different species of (Jaklitsch and Voglmayr, 2015). These sequences were aligned by MUSCLE software as implemented in MEGA X, and the trees were generated with the program IQ-TREE version 1.6.12. Branch support was assessed by a bootstrap analysis based on 1,000 pseudoreplicates.

### 2.6 Growth rate trials and morphological characterization

Following the method described in Zheng et al. (2021) with some modifications, growth rate and optimal growth temperature were determined on 90-mm-diameter Petri dishes on three different culture media: potato dextrose agar (PDA), corn meal dextrose agar (CMD; 40 g of cornmeal, 20 g of glucose, 18 g of agar, 1 L of distilled water), and synthetic low nutrient agar (SNA; 1 g of KH_2_PO_4_, 1 g of KNO_3_, 0.5 g of MgSO_4_, 0.5 g of KCl, 0.2 g of glucose, 0.2 g of sucrose, 18 g of agar, 1 L of distilled water) at 25°C, 30°C, and 35°C. Plugs of 6 mm diameter were extracted from the edge of 7-day-old PDA plates and placed approximately 1 cm from the border of the Petri dishes. Colony radii were measured after 24 h, 48 h, 72 h, 96 h, and 7 days. The time point when mycelium completely covered the surface of the plate was also recorded during this assay. Furthermore, the morphological characters of the colonies, such as their appearance, color, and spore production, were recorded at the same time. The development of T154 was also verified at 37.5°C and 40°C, to determine the maximum temperature at which it can grow.

The T154 isolate was cultured in PDA for the evaluation of the microscopic morphology, and it was incubated at 25°C for a 72–96-h period. Pictures were taken with a Nikon Eclipse E600 microscope connected to a Nikon DS-Fi3 digital camera.

Spore production was evaluated with four replicates of each of the following isolates, T154, *T. harzianum*, *T. atrobrunneum*, *T. guizhouense*, and *T. lentiforme* inoculated in PDA medium and incubated for 7 days at 25°C. Spores were collected by washing the Petri dish with distilled water and then filtering the spore suspension through a filter cloth. The spores were counted with a Neubauer chamber.

### 2.7 Antagonism assay in dual cultures

The antagonistic capacity of the T154 isolate was evaluated *in vitro* against the grapevine trunk disease pathogens *P. minimum*, *P. chlamydospora*, and *D. seriata* by performing dual culture tests. First, mycelial plugs (6 mm in diameter) of *P. minimum* and *P. chlamydospora* were obtained from the edge of 7-day-old PDA culture plates grown at 25°C. The plugs of these two pathogens were incubated for 14 days at 25°C in PDA to give them an advantage over *Trichoderma*, due to their slow growth rate. Subsequently, mycelial plugs (6 mm in diameter) of T154 were placed next to the pathogens, at a distance of 55 mm, from the edge of the cultures on 7-day-old PDA plates grown at 25°C. In the case of *D. seriata*, both mycelial plugs were placed at the same time on the plate at a distance of 55 mm since they showed a similar speed of development. *Phaeoacremonium minimum*, *P. chlamydospora*, and *D. seriata* were also incubated individually without the presence of *Trichoderma* under the same environmental conditions as the controls.

Dual cultures were incubated at 12°C in the dark to simulate the behavior of the T154–pathogen interaction at the time of pruning in the field, in which the pathogens have a greater possibility of penetrating the vine plant. Each control and *Trichoderma*/pathogen combination was replicated four times. Pathogen growth diameters were measured over a period of 30 to 40 days, depending on the development time required for each pathogen. The inhibition percentage caused by *Trichoderma* was calculated with the following equation (Úrbez-Torres et al., 2020):


$$Growth\;inhibition\;(\%)=\;\frac{\left(D2-D1\right)}{D2}\cdot 100$$

where D1 is the diameter of the pathogen mycelium grown in the presence of *Trichoderma* and D2 is the diameter of the pathogen mycelium grown alone in the control plate.

### 2.8 Compatibility of *Trichoderma carraovejensis* with pesticides and biostimulants

An evaluation of the resistance of *T. carraovejensis* against five pesticides and three biostimulants (commonly used in commercial wineries) was carried out in this experiment. This assay helps us to preliminarily identify which products could affect the *T. carraovejensis* strain and reduce its efficacy as a biological control agent.

The test was carried out in 90 mm diameter Petri dishes with PDA medium that was amended with the concentrations of each product indicated in **Table 1**. Pesticides and biostimulants were added to the PDA-melted medium at 45°C, the medium was homogenized in a magnetic agitator at 500 rpm, and 15 ml of the solution was poured into each Petri dish. Subsequently, inoculation of each plate was carried out by placing a 6-mm diameter mycelium plug from *T. carraovejensis* of a 7-day-old PDA culture. PDA plates without adding any product were used as control. Four repetitions per treatment were made. Plates were kept at room temperature (24°C) and measurements of the diameters of each treatment were made on the 2nd, 4th, and 7th days after inoculation.

**Table 1:** Active ingredients used in the trials, together with their form of presentation, application, commercial dose, and dose used.

Trade name and use	Active ingredient	Manufacturer	Presentation	Application	Commercial dose	Dose used
**Solfoxidante** (pesticide)	Sulfur 80%	Afepasa	Powder	Control of powdery mildew and phytopathogenic fungi	20–30 kg/ha	30 kg/ha
**Kdos** (pesticide)	Copper 35%	Certis	Powder	Control of pathogenic bacteria and fungi	300 g/hl	300 g/hl
**Azufre Micronizado P300/100** (pesticide)	Sulfur 98.5%	Afepasa	Powder	Control of powdery mildew, red spider mite, and eriophyids	20–30 kg/ha	30 kg/ha
**Heliosufre** (pesticide)	Sulfur 72% w/v	Agrichem	Liquid	Control of powdery mildew, red spider mites, and eriophyids	200–600 cc/hl	600 cc/hl
**Naturdai Nela** (pesticide)	Cinnamon extract	Idai Nature	Liquid	Powdery mildew control, mite repellency	Foliar application: 200–300 cc/hl	300 cc/hl
**Algafer** (biostimulant)	AA 4.5% + iron 5%	Idai Nature	Liquid	Natural biostimulant	Root application: 4–8 L/ha; foliar application: 200–400 cc/hl	300 cc/hl
**Brotaverd** (biostimulant)	Copper 1.6%–1.8% + manganese 0.75%–0.8% + zinc 0.5%–0.6%	Idai Nature	Liquid	Natural biostimulant	Root application: 3–5 L/ha; foliar application: 300–500 cc/hl	500 cc/hl
**Sergomil L60 ECO** (biostimulant)	Water-soluble copper (Cu) 5.5%; complexed copper (Cu) 2.8%	Servalesa	Liquid	Biostimulant with copper	150–300 cc/hl	300 cc/hl

### 2.9 Statistical analysis

All of the tests that were carried out were analyzed using IBM SPSS^®^ Statistics 21 (IBM Corp., Armonk, NY, USA). This software was used for the statistical analyses as follows: first, the Shapiro–Wilk test was used to check if there was normal distribution, then the homogeneity of variances was evaluated using Levene’s test, and one-way ANOVA was carried out to determine if there were significant differences. A *post-hoc* test (Duncan, *p*< 0.05) was performed to establish differences between groups.

## 3 Results

### 3.1 Phylogenetic analyses

**Figure 1:**
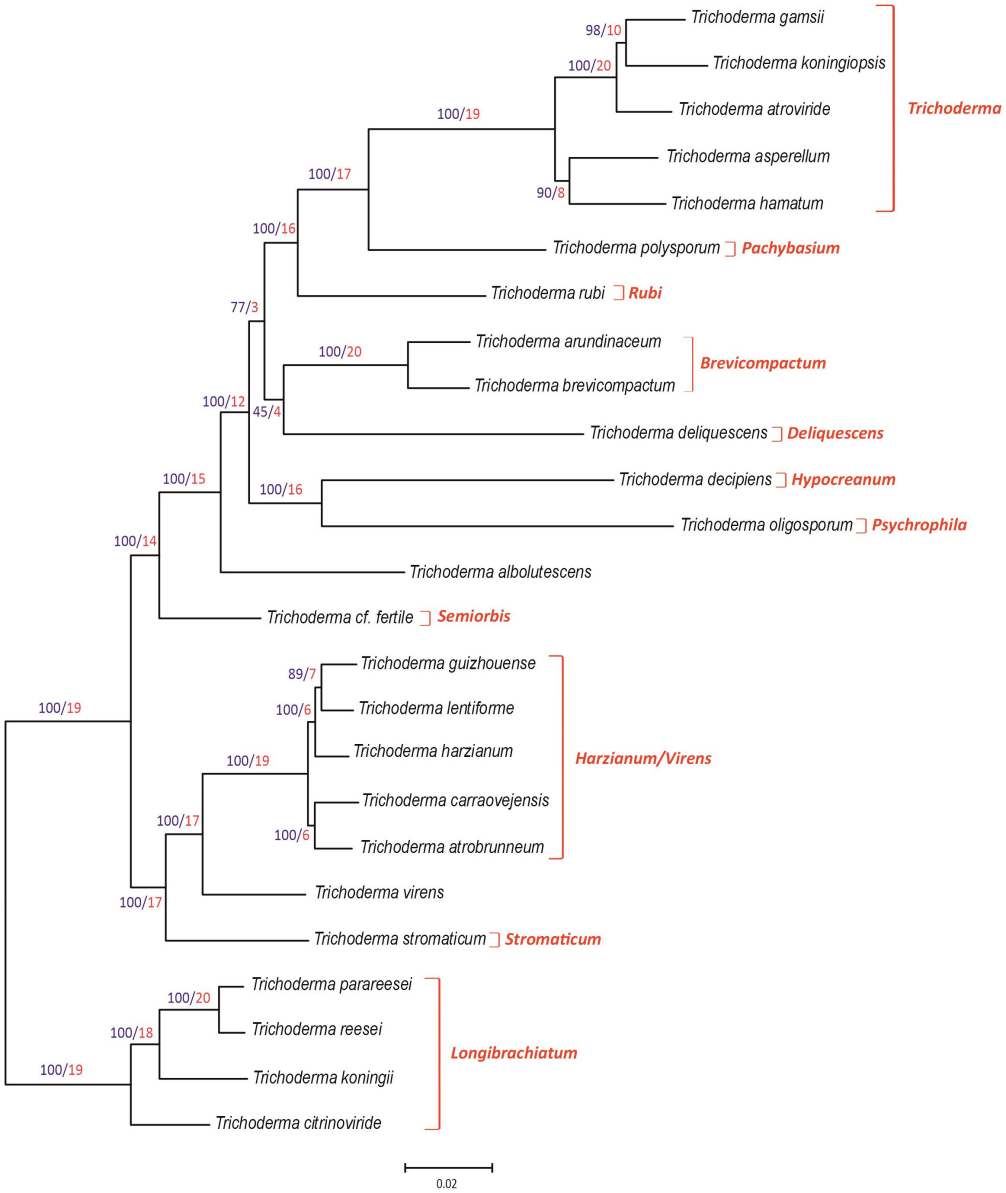
Phylogenic tree generated by the maximum likelihood analysis using concatenated sequences of 20 housekeeping genes of the genus *Trichoderma*. Sequences of housekeeping genes from *Trichoderma carraovejensis* T154 used for this study were deposited at the DDBJ/ENA/GenBank database under accession numbers indicated in **Supplementary Table S1**. Numbers on branches are bootstrap values in percentage (blue type) based on 1,000 pseudoreplicates and gene concordance factors (GCF, red type). GCF values indicate the number of each of the 20 independent trees that show the same branch, e.g., a value of 17 in a branch means that 17 out of the 20 individual trees show the same branch illustrated in the tree included in this figure. Lineage names, as previously described (Kubicek et al., 2019; Gutiérrez et al., 2021), are indicated in red type at the right of the tree.

The 25 *Trichoderma* species included in the phylogenetic analysis belong to 11 previously described lineages (Kubicek et al., 2019; Gutiérrez et al., 2021). To establish the position of the T154 isolate, we inferred a species tree based on a maximum likelihood analysis of concatenated alignments of 20 housekeeping genes that were retrieved in previous works from *Trichoderma* genome sequences corresponding to ex-type strains for each of the species used (Gutiérrez et al., 2021) as well as from the genome sequence of the T154 strain. In the resulting tree (**Figure 1**), our species of interest, T154, represents an individual branch within the *Harzianum/Virens* clade, with a bootstrap value of 100 and a GCF of 6. Therefore, these data support the assignment of the T154 isolate to a new species, which has been named *T. carraovejensis*. Furthermore, the topology of the phylogenetic tree was largely consistent with previously reported multispecies phylogenies for other *Trichoderma* species combinations (Jaklitsch and Voglmayr, 2015; Kubicek et al., 2019; Gutiérrez et al., 2021).

Three of the genetic markers (*acl1*, *tef1*, and *rpb2*) used in the study indicated above were analyzed separately. Thus, phylogenetic analyses of the *acl1*, *tef1*, and *rpb2* partial genes from a wider isolate representation resulted in individual trees that are consistent (**Supplementary Figures S1**-**S3**) with the main tree of the 20 housekeeping genes (**Figure 1**). In the three individual trees, the bootstrap values obtained on the *T. carraovejensis* branch were ≥85%.

### 3.2 Growth rate trials

**Figure 2:**
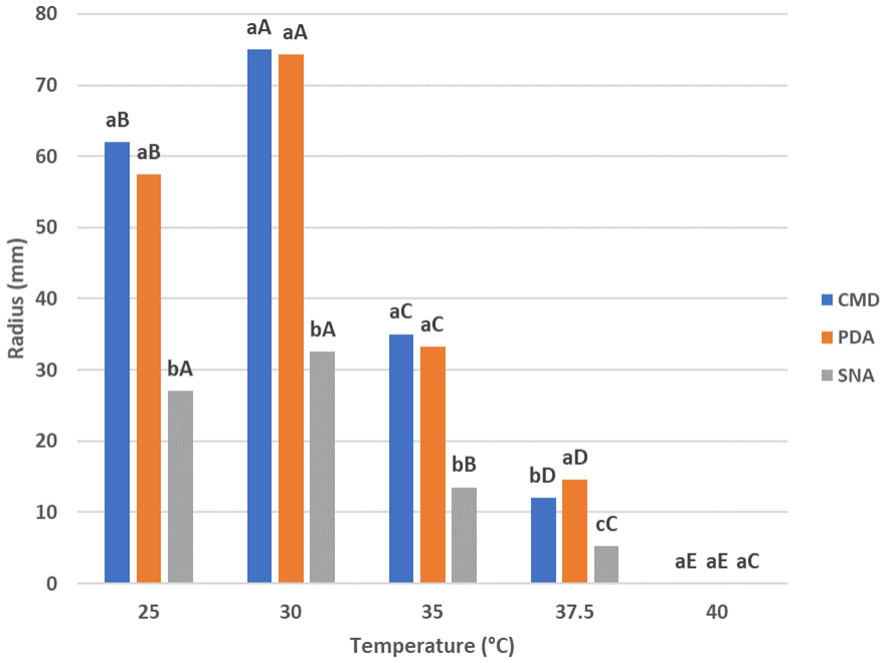
Growth rates of *Trichoderma carraovejensis* at 25°C, 30°C, 35°C, 37.5°C, and 40°C in potato dextrose agar (PDA), corn meal dextrose agar (CMD), and synthetic low-nutrient agar (SNA) media. Different lowercase letters indicate significant differences between culture media at the same temperature; Duncan test (*p* ≤ 0.05). Different capital letters indicate significant differences between temperatures in the same culture medium; Duncan test (*p* ≤ 0.05).

The results on the effect of different temperatures (25°C–40°C) on *T. carraovejensis* growth are summarized in **Figure 2**. The optimal growth temperature was estimated at 30°C for PDA and CMD culture media after 72 h from inoculation. For SNA, the optimal growth temperature varies between 25°C and 30°C, with no significant differences between these two temperatures. To conclude, the best media for *T. carraovejensis* growth are PDA and CMD, with no significant differences between them at 25°C, 30°C, and 35°C. However, there were significant growth differences between the first two media and SNA regardless of the temperature.

Furthermore, there was a significantly lower development of *T. carraovejensis* at 37.5°C in all media, as well as complete inhibition of fungal development at 40°C (**Figure 2**).

Further experiments were carried out in order to compare *T. carraovejensis* with the different species that were closely related in the phylogenetic trees: *Trichoderma harzianum* ULET87, *T. atrobrunneum* CECT 20730, *T. guizhouense* CECT 20731, and *T. lentiforme* CBS 100542. First, the growth at 25°C, 30°C, and 35°C was assessed in different culture media.

After 72 h of inoculation at 25°C on PDA, the colony of *T. carraovejensis* reaches a radius of 57.5 ± 1 mm, showing significant differences only with *T. harzianum*. Under the same conditions in CMD and SNA, *T. carraovejensis* growth does not differ significantly from the other species. A radius of 62 ± 1 mm in CMD and 27 ± 1 mm in SNA was reached. At 30°C after 72 h of growth from inoculation, *T. carraovejensis* demonstrated great growth rates in the different culture media used, standing out in the case of the PDA (74.5 ± 1 mm) and SNA (32.5 ± 1 mm) media above the rest of the species and in the case of the CMD (75 ± 1 mm) obtaining the highest growth rates, although with less difference with respect to the other species. In the case of PDA and SNA, a significant difference was observed in the development of *T. carraovejensis* with respect to the rest of the species. Under these conditions, *T. carraovejensis* completely covered the plate in CMD medium. At 35°C after 72 h of inoculation, *T. carraovejensis* reached a radius of 33.25 ± 1 mm in PDA, which is significantly higher than those reached by *T. harzianum* (25.25 ± 1 mm), *T. atrobrunneum* (14.25 ± 1 mm), and *T. guizhouense* (16.75 ± 1 mm) but identical to that reached by *T. lentiforme* (33.25 ± 1 mm). In CMD, *T. carraovejensis* also reaches one of the highest radii (35 ± 1 mm), being significantly higher than those reached by *T. harzianum* (17 ± 1 mm) and *T. atrobrunneum* (7 ± 1 mm). In the case of the SNA medium, *T. carraovejensis* reached a radius (13.5 ± 1 mm) that was significantly higher than those reached by *T. atrobrunneum* (7 ± 1 mm), *T. guizhouense* (8.5 ± 1 mm), and *T. lentiforme* (10.75 ± 1 mm), but significantly lower than that reached by *T. harzianum* (17 ± 1 mm). For most of the strains analyzed, a drastic decrease in growth was observed in all the media used when the growth was assessed at 35°C (**Supplementary Figure S4**).

**Figure 3:**
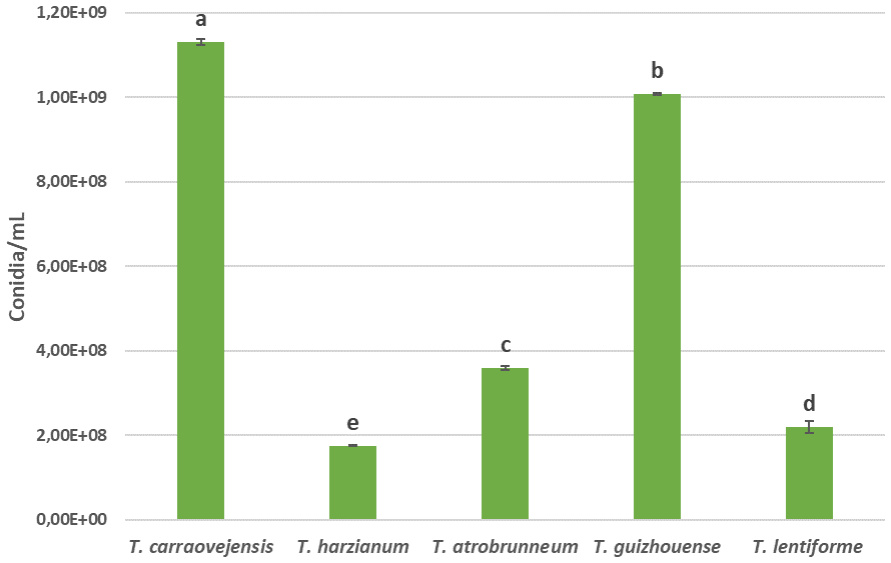
Spore production, expressed as conidia/mL, in 7-day cultures on PDA of all species compared. Different letters indicate significant differences between *Trichoderma* species. Duncan test (*p* ≤ 0.05).

Finally, a comparison between conidia production was performed in this experiment. An evaluation of the production of conidia was done 7 days after inoculation on PDA medium at 30°C. *Trichoderma carraovejensis* was able to produce 1.13 × 10^9^ conidia/ml, a higher value than the other species used for comparison: 1.75 × 10^8^ conidia/ml of *T. harzianum*, 3.59 × 10^8^ conidia/ml for *T. atrobrunneum*, 1.01 × 10^9^ conidia/ml of *T. guizhouense*, and 2.19 × 10^8^ conidia/ml for *T. lentiforme*, which represent 84.51%, 68.23%, 10.62%, and 80.62% less than *T. carraovejensis*, respectively (**Figure 3**).

### 3.3 Morphological characterization

**Figure 4:**
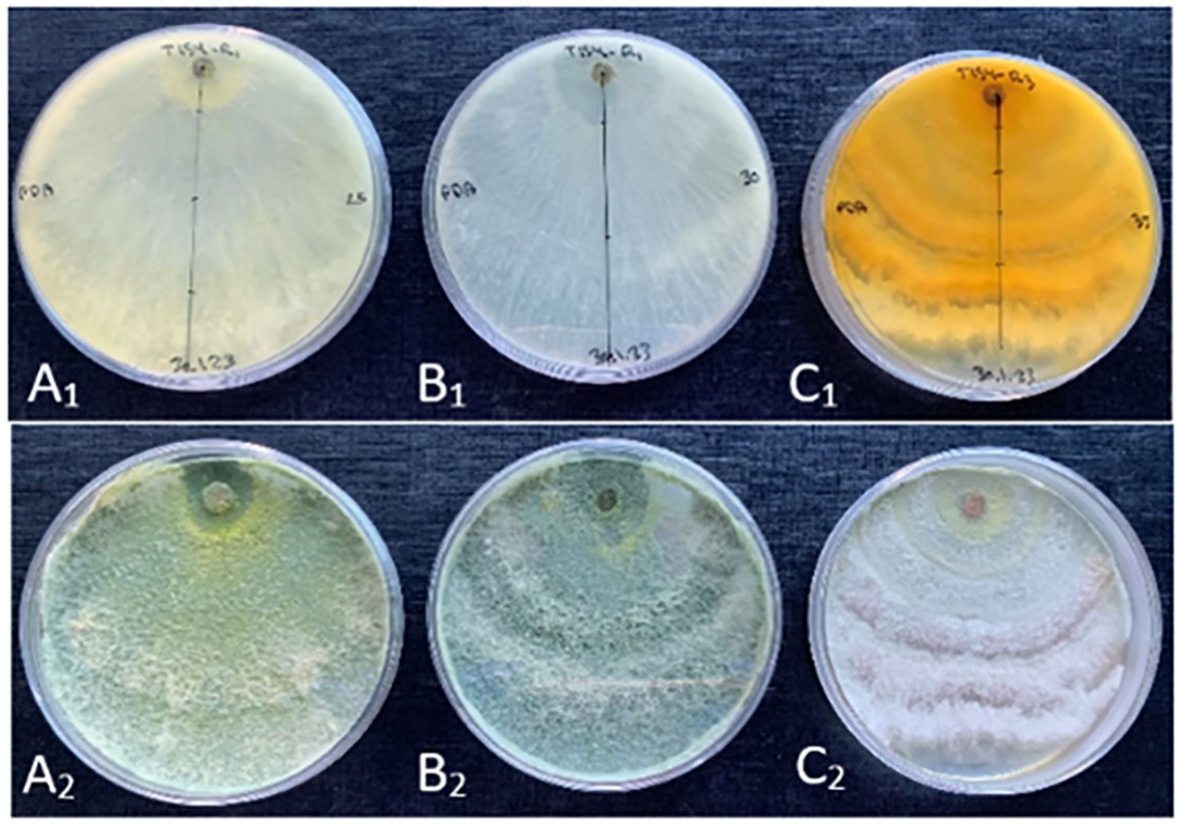
(**A_1_, B_1_, C_1_**) reverse side of the *Trichoderma carraovejensis* plate at 25°C, 30°C, and 35°C, respectively. (**A_2_, B_2_, C_2_**) obverse side of the *T. carraovejensis* plate at 25°C, 30°C, and 35°C, respectively.

Morphological characterization for the new species *T. carraovejensis* at three different temperatures showed different features (**Figure 4**). First, *T. carraovejensis* varied depending on the growth temperature. Development at 25°C gave rise to the appearance of yellow-green pigments. Second, at 30°C, the pigments were greenish tones, and finally at 35°C, yellow-orange tones could be observed at the base of the culture medium (**Figure 4**).

**Figure 5:**
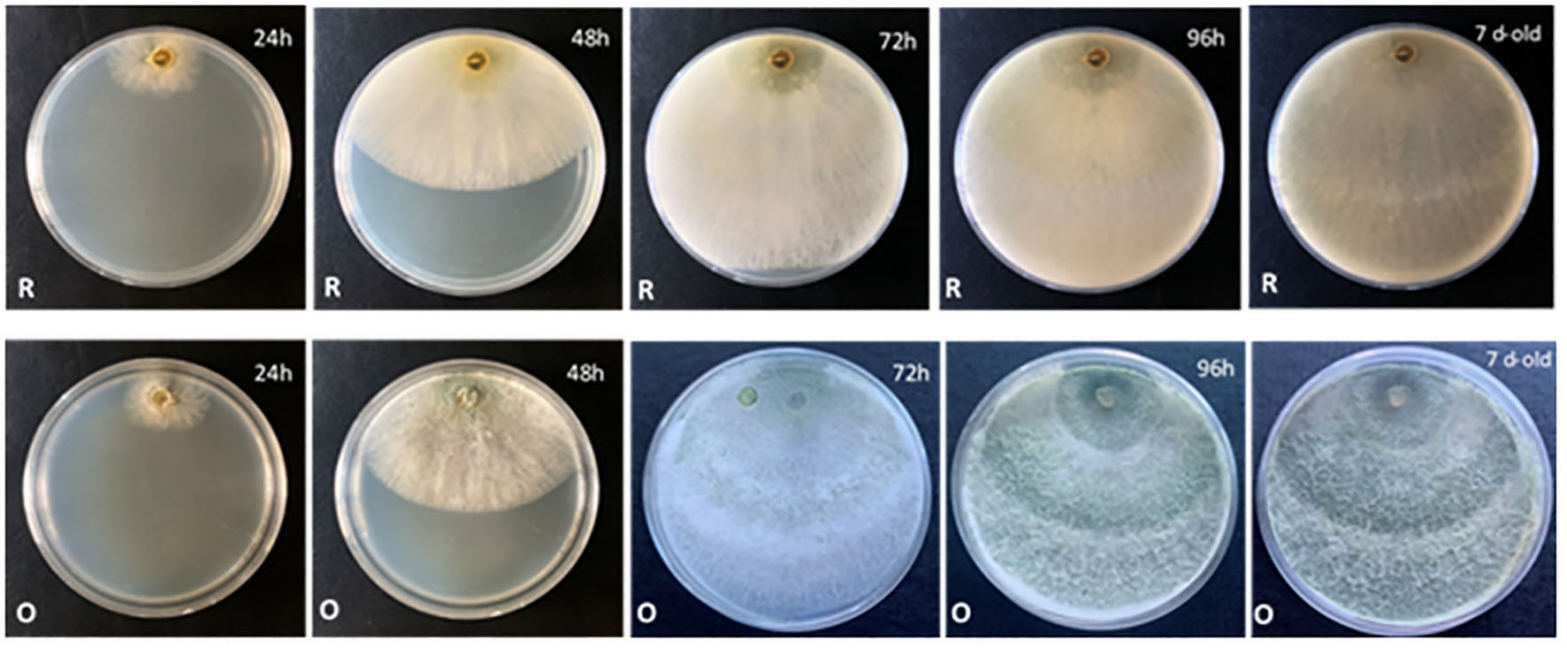
Morphology of *Trichoderma carraovejensis* in PDA at 30°C for 24 h, 48 h, 72 h, 96 h, and 7 days [reverse (R) and obverse (O) of the plate].

Thus, according to the results, the most useful culture medium for morphological comparison was PDA. At 30°C, all the compared species developed a growth pattern in concentric circles, with *T. carraovejensis* having the largest radius. At this temperature, *T. carraovejensis* began its development with a dense white mycelium. The first pigments to appear in the plate at 48 h were yellow tones, shortly after transforming to light greenish tones with subsequent darkening as the number of conidia in the plate increased. One of the main differences in comparison to the rest of the *Trichoderma* strains was observed after 7 days of growth when *T. carraovejensis* was the only one that no longer had areas with white mycelium. The entire plate was covered with conidia. This fact does not occur with the other species, which after 7 days continue to present alternate white mycelium zones between the conidia generation zones (**Figure 5**).

Comparison of morphological characterization was based on an *in-vitro* experiment at 35°C, which is the best range of temperature to differentiate *T. carraovejensis* from its closest *Trichoderma-*related species (**Supplementary Figure S5**)*. Trichoderma harzianum sensu stricto*, *T. atrobrunneum*, and *T. guizhouense* were not able to colonize more than half of the Petri dish plate in PDA, so this is a morphological criterion that can discriminate close species as different ones. If we compare *T. carraovejensis* and *T. lentiforme*, the whole plate was covered after 7 days in PDA at 35°C, and similar concentrical rings could be visualized and green pale olive colors were identified. However, from the point of inoculation, *T. lentiforme* had a yellowish and pale white color, which was also observed at the border of the plate where white color was identified. However, *T. carraovejensis* presented a homogeneous green pale color except at the point of inoculation where a strong green color was identified; possibly, it was due to the high concentration of conidia in comparison to the rest of *Trichoderma* species as confirmed in **Figure 3**.

### 3.4 Taxonomy

**Figure 6:**
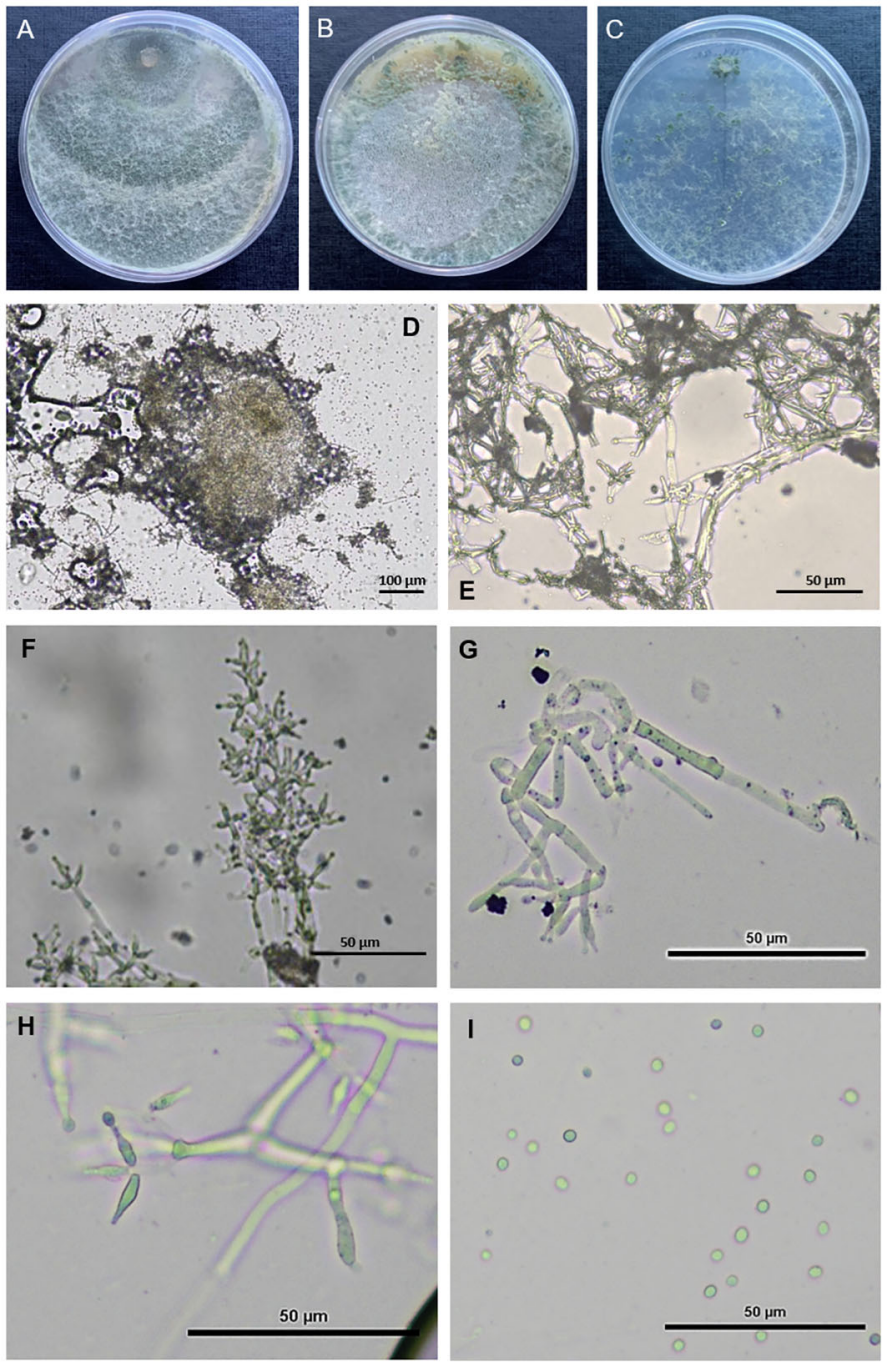
Observation of *Trichoderma carraovejensis*. **(A)** Cultures after 7 days at 30°C on PDA medium. **(B)** Cultures after 7 days at 30°C on CMD medium. **(C)** Cultures after 7 days at 30°C on SNA medium. **(D)** Pustules. **(E–H)** Conidiophores and phialides on PDA. **(I)** Conidia on PDA.

*T. carraovejensis*—G. Carro-Huerga, L. Zanfaño, S. Gutiérrez, P.A. Casquero Luelmo. sp. nov. (**Figure 6**).

***Etymology***—Referring to the winery where the isolate was obtained: “Pago de Carraovejas”.

***Typus***—Spain, Castilla y León, Valladolid, Peñafiel, grapevine bark of vine plant (*Vitis vinifera*), G. Carro-Huerga (GenBank accession number JAZAQE000000000).

Fast growing colonies. At the beginning of the development of the fungus, white cottony pustules were formed, which subsequently sporulated, taking on greenish colors and a granular texture. Pallid green reverse. Mycelium composed of branched, septate and hyaline hyphae. The conidiophores presented paired lateral branches and lageniform to utriform phialides, elongated in shape and broader at the base, appeared individually or in groups of two or three, (10.6–)12.0–12.2(–16.2) × (2.1–)2.8–2.9(–3.0) μm, length/width ratio. The globose-shaped conidia with smooth edges showed greenish tones, 2.1–2.5 × 2.6–2.8 μm, length/width ratio (**Figure 6**).

***Culture characteristics—*** Optimal growth temperature at 30°C. Colony radius on CMD after 72 h of growth: 62 ± 1 mm at 25°C, 75 ± 1 mm at 30°C, and 35 ± 1 mm at 35°C, covering the plate after 3 days at 30°C. Translucent mycelium at 48 h, appearance of the first yellow-green tones at 96 h with the formation of spores. Dense and greenish mycelium at 7 days with greater spore production at the edges of the plate. Colony radius on PDA after 72 h: 57.5 ± 1 mm at 25°C, 74.25 ± 1 mm at 30°C, and 33.25 ± 1 mm at 35°C, covering the plate after 4 days at 25°C and 30°C. Formation of dense mycelium in concentric circles. Appearance of the first spores at 48 h around the sowing disc. Green pigments that increase in intensity as the days pass since inoculation, which corresponds to an increase in the number of spores produced. Colony radius on SNA after 72 h: 27 ± 1 mm at 25°C, 32.5 ± 1 mm at 30°C, and 13.5 ± 1 mm at 35°C, covering the plate after 7 days at 25°C and 30°C. Formation of a sparse mycelium with greenish pigments after 48 h. The production of spores in PDA at 7 days was 1.13 × 10^9^ spores/ml.

***Notes***—In this study, *T. carraovejensis* was isolated from grapevine bark. *Trichoderma carraovejensis* was differentiated from other species by phylogenetic analysis of the sequences of 20 housekeeping genes from 24 different *Trichoderma* species and by morphological comparison with *T. harzianum*, *T. atrobrunneum*, *T. guizhouense*, and *T. lentiforme*, the species closest to it in the generated phylogenetic tree.

### 3.5 Antagonism assay in dual cultures

**Figure 7:**
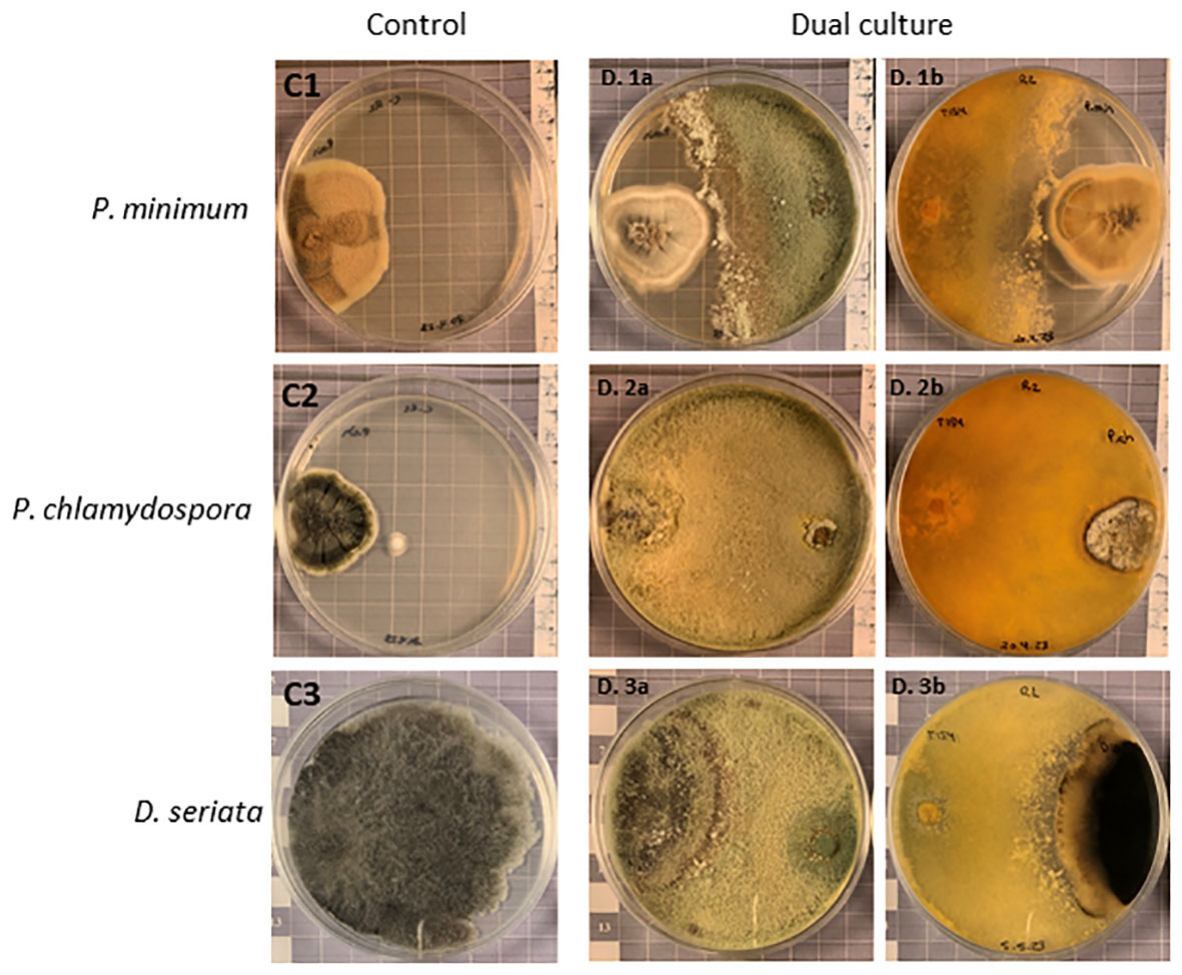
Dual culture antagonism experiment. The C1, C2, and C3 plates show *Phaeoacremonium minimum*, *Phaeomoniella chlamydospora*, and *Diplodia seriata* controls, respectively. D. 1a and D. 1b show antagonistic activity of *T. carraovejensis* against *P. minimum* and D. 2a and D. 2b show antagonistic activity of *T. carraovejensis* against *P. chlamydospora* after 38 days of growth on PDA at 12°C, and D. 3a and D. 3b show antagonistic activity of *T. carraovejensis* against *D. seriata* after 31 days of growth on PDA at 12°C.

The levels of antagonism of *T. carraovejensis* against the GTD pathogens *P. minimum* and *P. chlamydospora* were evaluated after 38 days of growth and for *D. seriata* after 31 days. This was due to the slow development of the isolates at 12°C. The mean percentages of radial growth inhibition (RI) for *P. minimum*, *P. chlamydospora*, and *D. seriata* were 21.04%, 15.34%, and 34.08%, respectively. *Trichoderma carraovejensis* exhibited the best inhibition values against the pathogen *D. seriata* when assays were carried out at 12°C (**Figure 7**).

*Trichoderma* was able to stop the development of *P. minimum* but without colonizing it. In the case of *P. chlamydospora*, *T. carraovejensis* stopped the growth of the pathogen, and the biocontrol agent was able to colonize the pathogenic fungus, growing and producing spores on it. As for *D. seriata*, a clear arrest of the development of the pathogen and an overgrowth of *Trichoderma* on it were observed.

### 3.6 Compatibility of *Trichoderma carraovejensis* with pesticides and biostimulants

**Figure 8:**
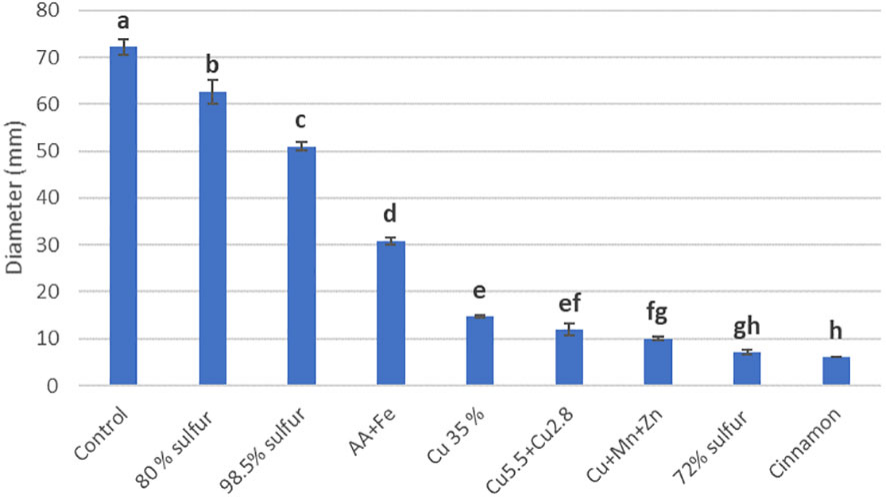
Growth diameter of *Trichoderma carraovejensis* in the media with the different products used 2 days after inoculation. Different letters indicate significant differences between the products used. Duncan test (*p* ≤ 0.05).

**Figure 9:**
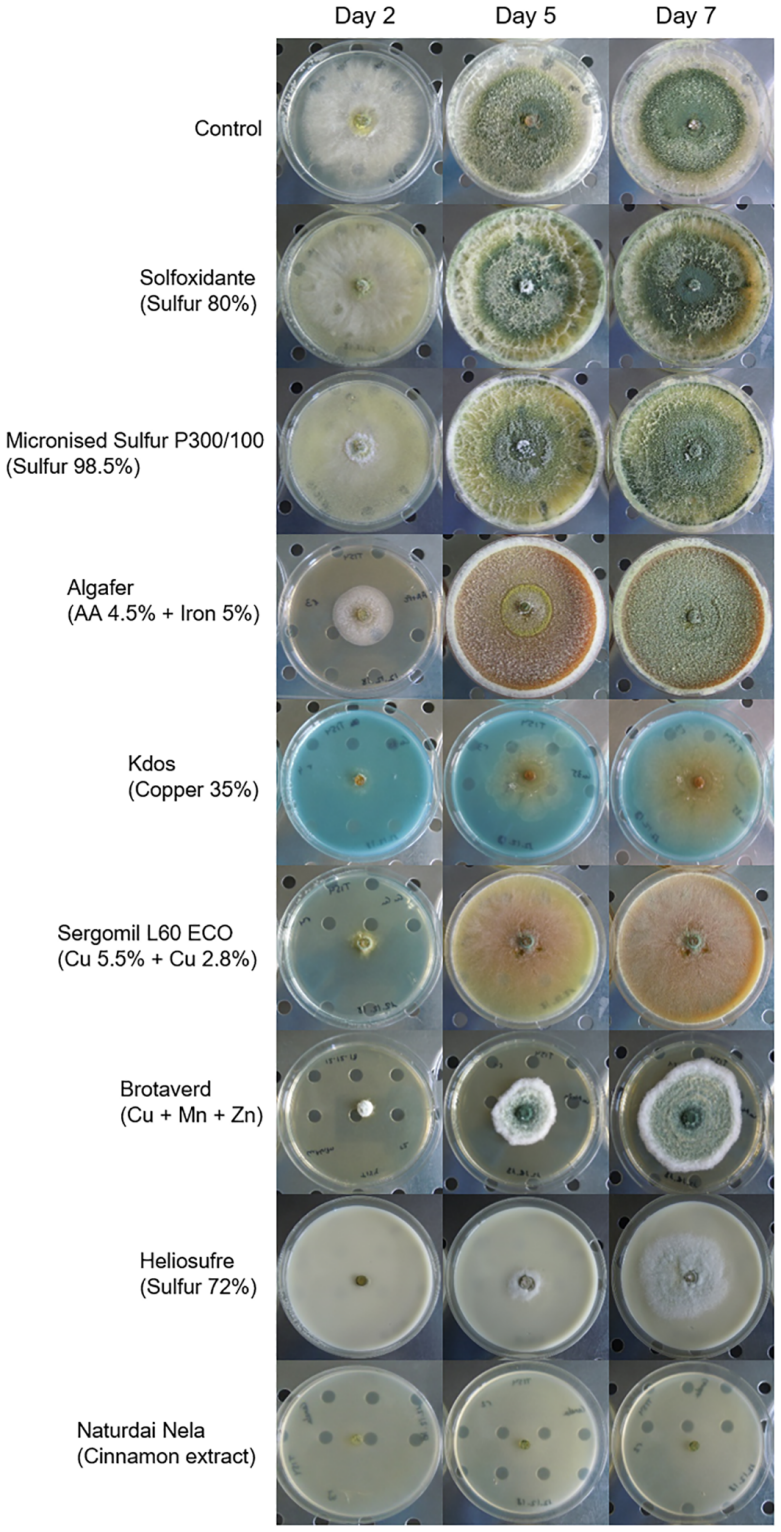
Development of *Trichoderma carraovejensis* 2, 5, and 7 days after inoculation in the culture media with the different pesticides and biostimulants used in the trial.

Assays performed with *T. carraovejensis* grown in the presence of different products showed significant differences (*p*< 0.05) among pesticides and biostimulants. The colony diameter in the control plate (=*Trichoderma* growing alone in PDA medium) reached the highest value, 72.25 ± 1 mm, and this value was significantly higher in comparison to the rest of the tested products. *Trichoderma carraovejensis* demonstrated the greatest compatibility with the 80% sulfur-based product (62.75 ± 1 mm) and differed significantly to 98.5% sulfur (51.00 ± 1 mm) and the products described in **Table 1**. *Trichoderma* reached a diameter of 30.88 ± 1 mm in combination with the product based on amino acids and iron. Very similar values were obtained with the copper-based products, with no significant differences between Cu 35% (14.75 ± 1 mm) and Cu5.5 + Cu2.8 (11.88 ± 1 mm) and between the latter and Cu+Mn+Zn (10.00 ± 1 mm). No significant differences were also observed between Cu+Mn+Zn and 72% sulfur (7.13 ± 1 mm). Cinnamon showed the lowest compatibility with *T. carraovejensis*, inhibiting its growth (**Figures 8**, **9**).

## 4 Discussion

In this study, a new autochthonous *Trichoderma* species, isolated from grapevine plants in Castilla y León (Spain), has been characterized and described. A multigene phylogenetic analysis was performed based on 20 housekeeping genes (**Supplementary Table S1**). The phylogenetic analysis led to the conclusion that *T. carraovejensis* maps inside the *Harzianum/Virens* clade (Jaklitsch and Voglmayr, 2015). Within this clade, *T. carraovejensis* is close to *T. guizhouense*, *T. lentiforme*, *T. harzianum sensu stricto*, and *T. atrobrunneum. Trichoderma carraovejensis* has a morphology typical of species belonging to this clade, with lageniform to lectiform phialides and globose to subglobose conidia (Chaverri et al., 2015). This study is complemented by three other phylogenetic analyses, using *acl1*, *rpb2*, and *tef1* partial sequences from a plethora of *Trichoderma* species, as recommended in the ICCT protocols. The results are consistent with those obtained in the multigene phylogenetic analysis. This is an extended evaluation where 20 complete housekeeping genes are evaluated instead of using partial housekeeping genes where great effort is put forth to verify the discovery of a new species.

In terms of microscopic morphological comparison, *T. carraovejensis* presents lageniform to lectiform phialides in comparison to *T. guizhouense* which has phialides mostly in whorls (Korkom and Yıldız, 2023). *Trichoderma harzianum sensu stricto* presents phialides ampulliform to lageniform and conidia globose and subglobose and is smaller in comparison to *T. carraovejensis.* However, both of them have pyramidal conidiophores. If *Trichoderma atrobruneum* is compared with *T. carraovejensis*, both of them present widely spaced branches, terminating in a whorl of two–five phialides, and have a similar shape and form of conidiophores so it is necessary to use a genetic identification tool (Jang et al., 2018). *Trichoderma lentiforme* presents phialides ampulliform, sharply constricted below the tip to form a narrow neck which is pretty similar to our *T. carraovejensis* species apart from having a similar shape and form (Chaverri et al., 2015). Thus, a genetic identification is necessary to confirm the real differences.

The description of this new species has also been based on the comparison of growth and development rates versus species mapping close to *T. carraovejensis* in the phylogenetic trees (*T. guizhouense*, *T. lentiforme*, *T. harzianum*, and *T. atrobrunneum*). This study includes comparative phylogenetic data analysis and also evaluates morphological characters, as recommended, in order to totally differentiate *Trichoderma* strains (Li et al., 2013). Thus, according to the genetic, morphological, growth rate, and spore production differences, it was possible to confirm a new species that has been described in this complex clade (Druzhinina et al., 2010).

Spore production is an important character in the selection of a new potential biocontrol species since all of the *Trichoderma*-based products are presented as spore suspensions (Woo et al., 2014). In the present case, *T. carraovejensis* produces a high and significantly different number of spores in comparison to its neighbor species, especially *T. harzianum sensu stricto*, which has been described for mass production (Mahamud, 2019). This is another parameter that would allow us to easily produce *Trichoderma* spores at a large scale.

The optimal growth temperature of *T. carraovejensis* is 30°C. At this temperature, *T. carraovejensis* shows a significantly higher growth rate than the rest of the species, coinciding with previous reports where strains of this clade are adapted to warmer climates (Harman and Kubicek, 1998b). In general, the development of *T. carraovejensis* is higher in all temperatures than that of the rest of the species used in this comparison, with values very similar in some cases to *T. atrobrunneum*, a very common species in Southern Europe (Chaverri et al., 2015). Another point to consider is the growth of *Trichoderma* at the human body temperature, approximately 35°C and 37.5°C (Geneva et al., 2019). There are several studies that identify *Trichoderma* as a human pathogen (Hatvani et al., 2013) due to this, and growth tests were carried out at 37.5°C and 40°C to verify the development of *T. carraovejensis* at these temperatures. At 37.5°C, a notable decrease in the development of *Trichoderma* was observed in all the media used, not exceeding a 30-mm radius after 21 days and not being able to produce spores. At 40°C, *T. carraovejensis* completely inhibits its development. These data would also discard *T. carraovejensis* as a human or mammal pathogen. Despite this, caution should be taken when these microorganisms are used in agriculture (Kredics et al., 2021). Security for humans is an important concern as several studies place the origin of the identified *Trichoderma* isolates in human infections in the environment (Hatvani et al., 2019).

*Trichoderma carraovejensis* has been isolated from grapevine wood in a plot in Ribera del Duero PDO (protected designation of origin) (Spain) (Carro-Huerga et al., 2020). Regarding its potential as a BCA, the activity of this species against three fungi that cause GTDs was evaluated. *Phaeoacremonium minimum* and *P. chlamydospora* are considered the pioneer fungi of esca disease and the main pathogen of Petri disease is *D. seriata*, causing *Botryosphaeria* dieback (Mondello et al., 2018). It is well known that one of the routes of entry for the microorganisms that cause GTD is pruning wounds (Gramaje et al., 2018); thus, experiments were carried out in winter when daytime temperatures are significantly lower than the optimum temperature for *Trichoderma* growth. For this reason, a dual test was carried out at 12°C (Carro-Huerga et al., 2021). This temperature was used to simulate field conditions during pruning seasons, in order to analyze the behavior and development of both the *Trichoderma* and the pathogens. *Trichoderma*, *P. minimum*, *P. chlamydospora*, and *D. seriata* have shown a very slow development, requiring 38 days for *P. minimum* and *P. chlamydospora* and 31 days for *D. seriata* growth after inoculation to obtain conclusive data regarding the inhibition of growth of these pathogens by *Trichoderma*. The best results were obtained for *D. seriata*, with an inhibition percentage of 34.08%, followed by *P. minimum* with 21.04% inhibition, and the lowest value was obtained by *P. chlamydospora* with 15.34%. These data show a lower relevance of mycoparasitism observed by *Trichoderma* compared with other studies, where inhibition data are collected approximately 5 days after inoculation (Mayo et al., 2015; Porteous-Álvarez et al., 2023). This could most probably be due to the low temperature used, below the optimal for *T*. *carraovejensis* growth. Moreover, this *Trichoderma* strain has already been proven as an effective biological control agent used as a protective method in pruning wounds and fungal pathogens of GTDs. Firstly, assays in *Trichoderma–*plant interaction proved that this strain (*T. carraovejensis*, T154) is able to persist during winter conditions from November to February in the pruning wounds of vine plants, and the re-isolation percentage was up to 85%–90%. Also, during this experiment, this *Trichoderma* strain was the only colonizer in agar plates from the vine chips sown; however, it was not able to perform colonization far from the point of inoculation (Carro-Huerga et al., 2021). Secondly, *T. carraovejensis* T154 performed a good control of one of the main pathogens of fungal GTDs (*P. minimum*). *Trichoderma*–pathogen interaction showed a mycoparasitism mode of action after being analyzed in microscopy assays as well as spore adhesion and hyphal adhesion as mechanisms proven in this interaction. Finally, a triple interaction (*Trichoderma*–plant–pathogen) was analyzed using confocal laser scanning microscopy (CSLM) and scanning electron microscopy (SEM). In this experiment, *T. carraovejensis* T154 was able to colonize pruning wounds without causing any damage to the vine plant, and the mechanism of action was niche exclusion. In addition to this test, *T. carraovejensis* was evaluated as the fungus that was able to colonize pruning wounds in xylem vine tissues (Carro-Huerga et al., 2020). However, under field conditions, the triple interaction (*Trichoderma*–pathogen–plant interaction) needs to be evaluated for a longer period of time.

The inhibition results obtained for *D. seriata* did not greatly differ from those obtained in similar tests with other *Trichoderma* species, even though those were mostly carried out at temperatures approximately 25°C (Blundell et al., 2021; Pollard-Flamand et al., 2022). *Trichoderma carraovejensis* colonizes the entire Petri dish during this 31-day period, stopping the growth of *D. seriata*. Inhibition values of *P. minimum* were slightly higher than those shown in other studies carried out at higher temperatures (Carro-Huerga et al., 2023), which could guarantee a higher efficacy of *T. carraovejensis* under field conditions during the pruning season against this grapevine pathogen. In the case of *P. chlamydospora*, although the percentage of inhibition was low, **Figure 7** shows how *T. carraovejensis* was able to colonize the entire plate and even overgrew the pathogen. This experiment showed that *T. carraovejensis* was able to stop the pathogen from growing in a simulated laboratory test in cold conditions. Moreover, this strain was able to overgrow *D. seriata*. Therefore, *T. carraovejensis* is a potential preventative method against the main fungal pathogens that cause GTDs. However, it is necessary to evaluate this strain under field conditions to confirm its efficacy in terms of colonization.

Combined testing of biological control agents and pesticides or biostimulants may result in increased efficacy of these compounds to control and eradicate fungal diseases (Amaresh et al., 2019). This combination could reduce the doses of pesticides required for the field. Moreover, it is important to study this compatibility in order to facilitate colonization of vines and its persistence in time by *Trichoderma*. Thus, a lesser number of applications could allow vine growers to reduce costs in terms of the number of spraying per year as well as reduce the cost of bioproducts. For this purpose, it is necessary to study the compatibility of BCA with pesticides and biostimulants available on the market. *Trichoderma carraovejensis* showed high compatibility with the sulfur-based products Solfoxidante and micronized sulfur P300/100. Our strain shows a high compatibility with sulfur powder. This is a great advantage in the case of a combined use of *Trichoderma*–sulfur in the field, as sulfur powder is one of the most widely used products in vineyards (Bravo et al., 2023). In the case of Heliosufre (liquid sulfur), *Trichoderma* had its growth affected. However, nowadays, it is uncertain why sulfur powder has a significantly less negative effect than liquid sulfur, so this is an interesting point to unravel. As shown in one study, a strategy consisting of a combination of sulfur and *T. afroharzianum* strain NAIMCC-F-01938 could be an option for biocontrolling diseases (Sawant et al., 2017). It is important to highlight that this strain also belongs to the same clade, so it is interesting to study the genetic implications associated with the resistance to this active substance. Some studies show that the combined use of algae and *Trichoderma* has a beneficial effect on crop development (Sani et al., 2022). In the case of Algafer, a product that combines algae, amino acids, and iron, a slowing down of *Trichoderma* development is observed. This fact could be due to the type of algae used in the commercial product or due to its formulation. Copper can be used as a fertilizer and fungicide. Some studies have shown some isolates of *T. harzianum* that sporulated to a greater extent in the presence of copper (Banik and Pérez-de-luque, 2017) and how other *Trichoderma* species were able to tolerate this element accumulating it on the surface of the cell wall (Ladi et al., 2020). In other studies, however, it was observed that *Trichoderma* was affected by the presence of copper (Mayo-Prieto et al., 2022) such as in this work where a significant reduction in the development of *T. carraovejensis* in the presence of this element was observed. It is important to highlight that this strain is very sensitive to copper products, so it is not advisable to spray if preventative-based copper products are sprayed in winter. In order to improve its persistence, bioformulation and bioencapsulation assays are needed to have a good result in terms of durability and survival according to Singh et al. (2007) and Preininger et al. (2018). Cinnamon also showed antifungal activity against pathogenic fungi (Rusin et al., 2020). The combination of this product and *Trichoderma* completely inhibited its growth, so it is not advisable to spray cinnamon over vine plants if *Trichoderma* is present in the vineyard. *Trichoderma carraovejensis* shows some tolerance to several of the fungicides used in this trial. As observed in other studies such as Ramanagouda and Naik (2021), our *Trichoderma* strain and other indigenous species like *T. asperelloides*, *T. asperellum*, and *H. lixii* could be used for the integrated control of grapevine trunk diseases in their regions of origin. This approach is a preventative method for protecting pruning wounds of vine plants against GTDs.

## 5 Conclusion

In the present study, we have concluded, based on extensive and diverse phylogenetic analyses, that the vineyard soil isolate T154 corresponded to a new species, which has been named *T. carraovejensis*. The different experimental approaches used in this study and in previous studies allowed us to present this species as a new and promising option for the control of GTDs. Thus, *T. carraovejensis* exhibits a good adaptation to different temperatures, has great potential as a preventative method in pruning wounds against GTDs, and also has a high degree of compatibility with diverse chemical products used for this purpose.

## Data availability statement

The datasets presented in this study can be found in online repositories. Housekeeping genes of *T. carraovejensis* T154 generated during the present study are deposited in the NCBI/Bankit/GenBank repository under the accession numbers listed in the **Supplementary Table S1**.

## Author contributions

LZ: Writing – original draft, Validation, Software, Methodology, Investigation, Formal analysis, Data curation, Writing – review & editing, Visualization. GC-H: Writing – review & editing, Writing – original draft, Visualization, Validation, Software, Methodology, Investigation. ÁR-G: Writing – review & editing, Visualization, Validation, Software, Methodology, Investigation. SM-P: Writing – review & editing, Visualization, Validation, Software, Methodology, Investigation. RC: Writing – review & editing, Visualization, Validation, Software, Methodology, Investigation. SG: Writing – review & editing, Visualization, Supervision, Resources, Project administration, Funding acquisition, Conceptualization. PC: Writing – review & editing, Visualization, Supervision, Resources, Project administration, Funding acquisition, Conceptualization.
