# Supplementary material for: Trichoderma carraovejensis: a new species from vineyard ecosystem with biocontrol abilities against grapevine trunk disease pathogens and ecological adaptation
Source: Front Plant Sci. 2024 May 21;15:1388841. doi: 10.3389/fpls.2024.1388841 (PMC11148300; doi:10.3389/fpls.2024.1388841)
Supplement: Supplementary file 1 [file DataSheet_1.docx]

Supplementary Material

**Supplementary Table S1.** GenBank accession numbers of housekeeping genes of *Trichoderma carraovejensis* T154.

| **Gene** | **Predicted gene function** | **Accession number** |
| --- | --- | --- |
| *cpr1* | Cytochrome P450 reductase | OR757277 |
| *dpa1* | DNA polymerase alpha subunit | OR757278 |
| *dpd1* | DNA polymerase delta subunit | OR757279 |
| *erg1* | ergosterol monooxygenase/oxidase | OR757280 |
| *fas1* | Fatty acid synthase alpha subunit | OR757281 |
| *fas2* | Fatty acid synthase beta subunit | OR757282 |
| *his3* | Histone H3 | OR757283 |
| *lae1* | Gobal regulatory Gene/ chromatin methyl transferase | OR757284 |
| *lcb1* | Sphinganine palmitoyl transferase subunit 1 | OR757285 |
| *lcb2* | Sphinganine palmitoyl transferase subunit 2 | OR757286 |
| *mcm7* | DNA replication licensing factor | OR757287 |
| *pgk1* | Phosphoglycerate kinase | OR757288 |
| *rpb1* | RNA polymerase largest subunit | OR757289 |
| *rpb2* | RNA polymerase 2nd largest subunit | OR757290 |
| *sph1* | Sphinganine N acyl transferase subunit 1 | OR757291 |
| *top1* | Topoisomerase | OR757292 |
| *tps1* | Trehalose phosphate synthase | OR757293 |
| *tsr1* | Ribosomal biogenesis protein | OR757294 |
| *tub1* | Tubulin alpha subunit | OR757295 |
| *ubt1* | Ubiquitin thiolesterase | OR757296 |


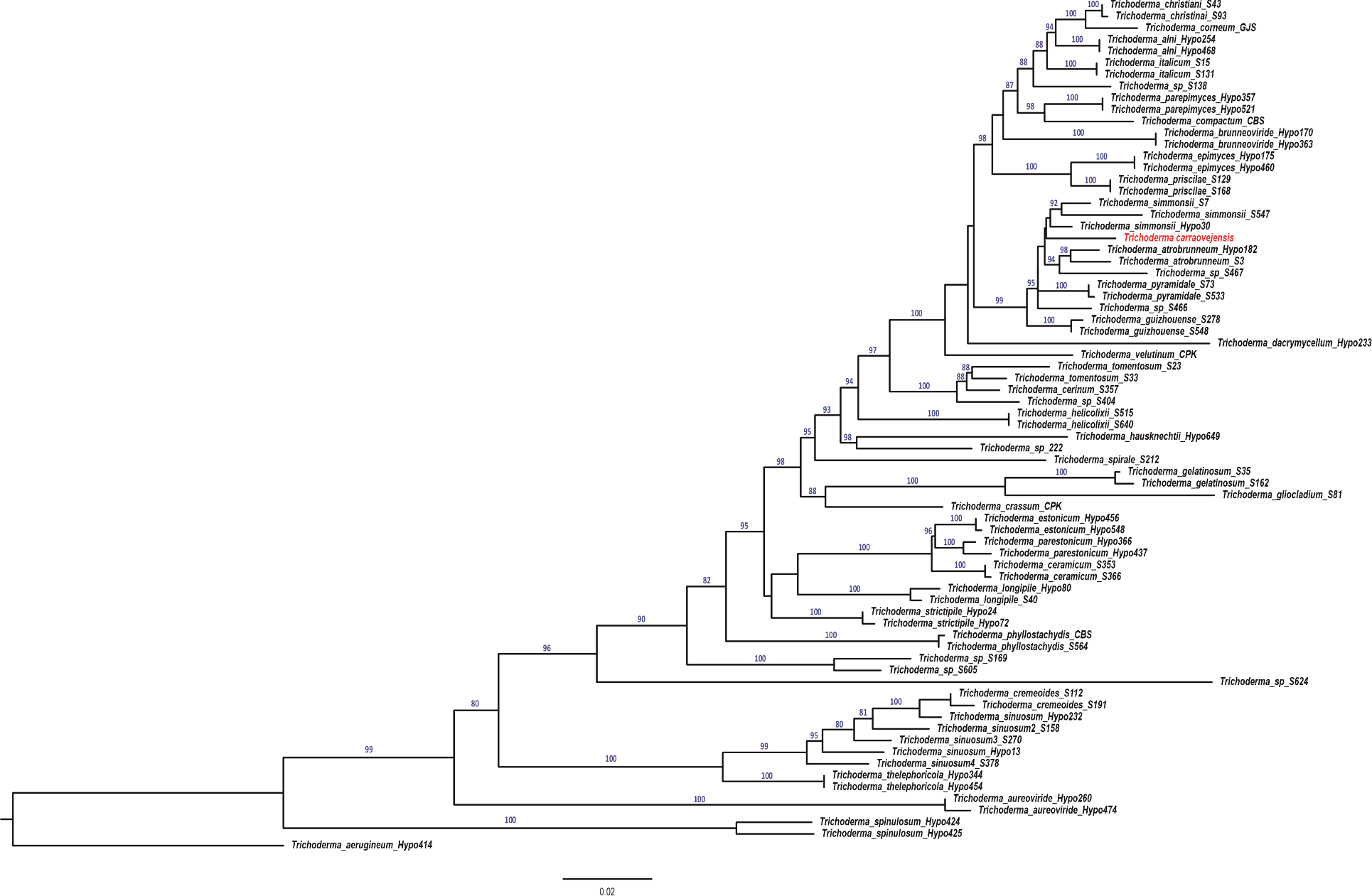


Supplementary Figure S1. Phylogenic tree of the genetic marker *acl1* (ATP citrate lyase) using partial amino acid sequences. The sequences were retrieved from different species of Jaklitsch and Voglmayr, 2015.


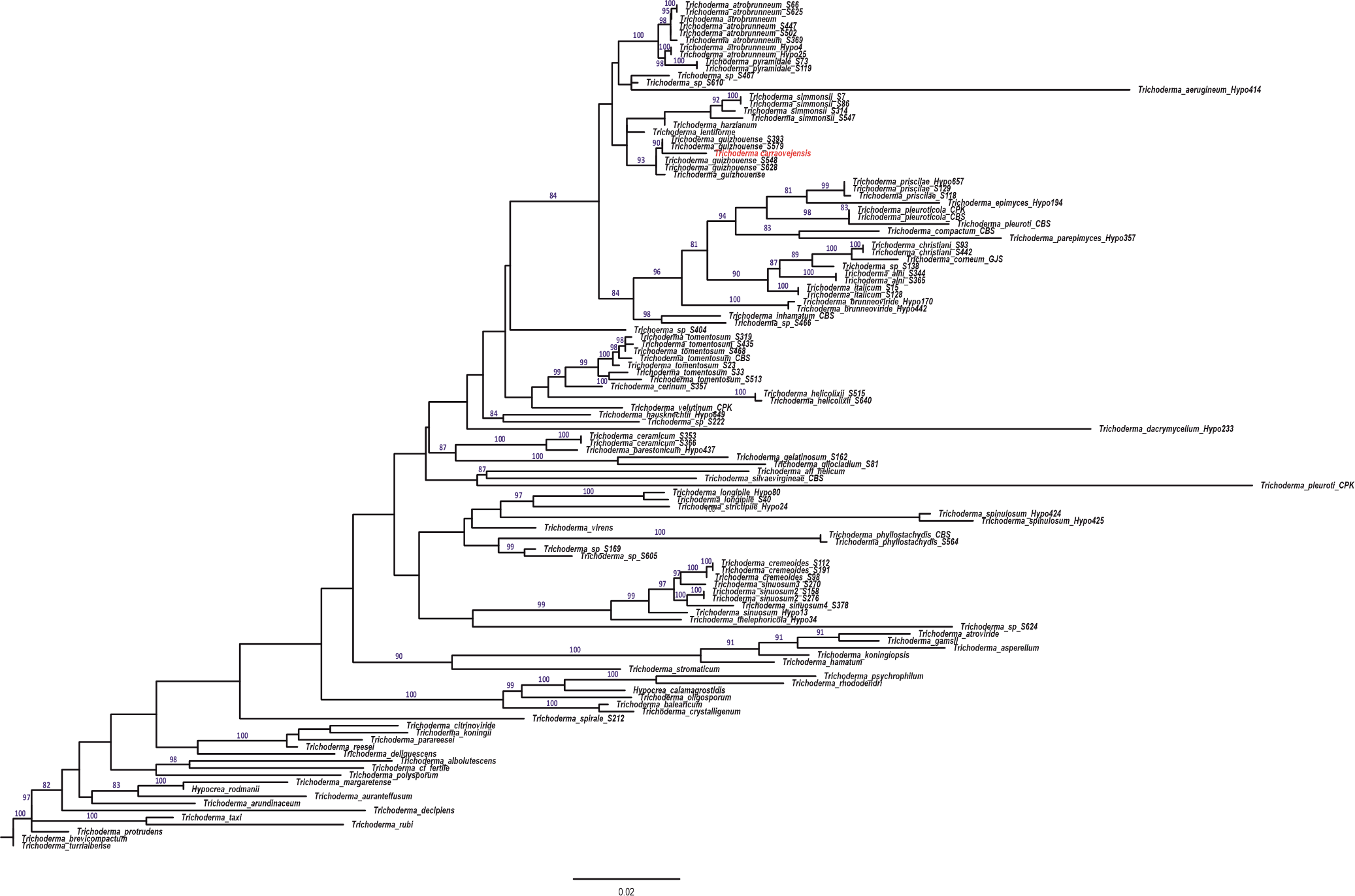


Supplementary Figure S2. Phylogenic tree of the genetic marker *tef1* (translation elongation factor 1-alpha) using partial amino acid sequences. The sequences were retrieved from different species of Jaklitsch and Voglmayr, 2015.


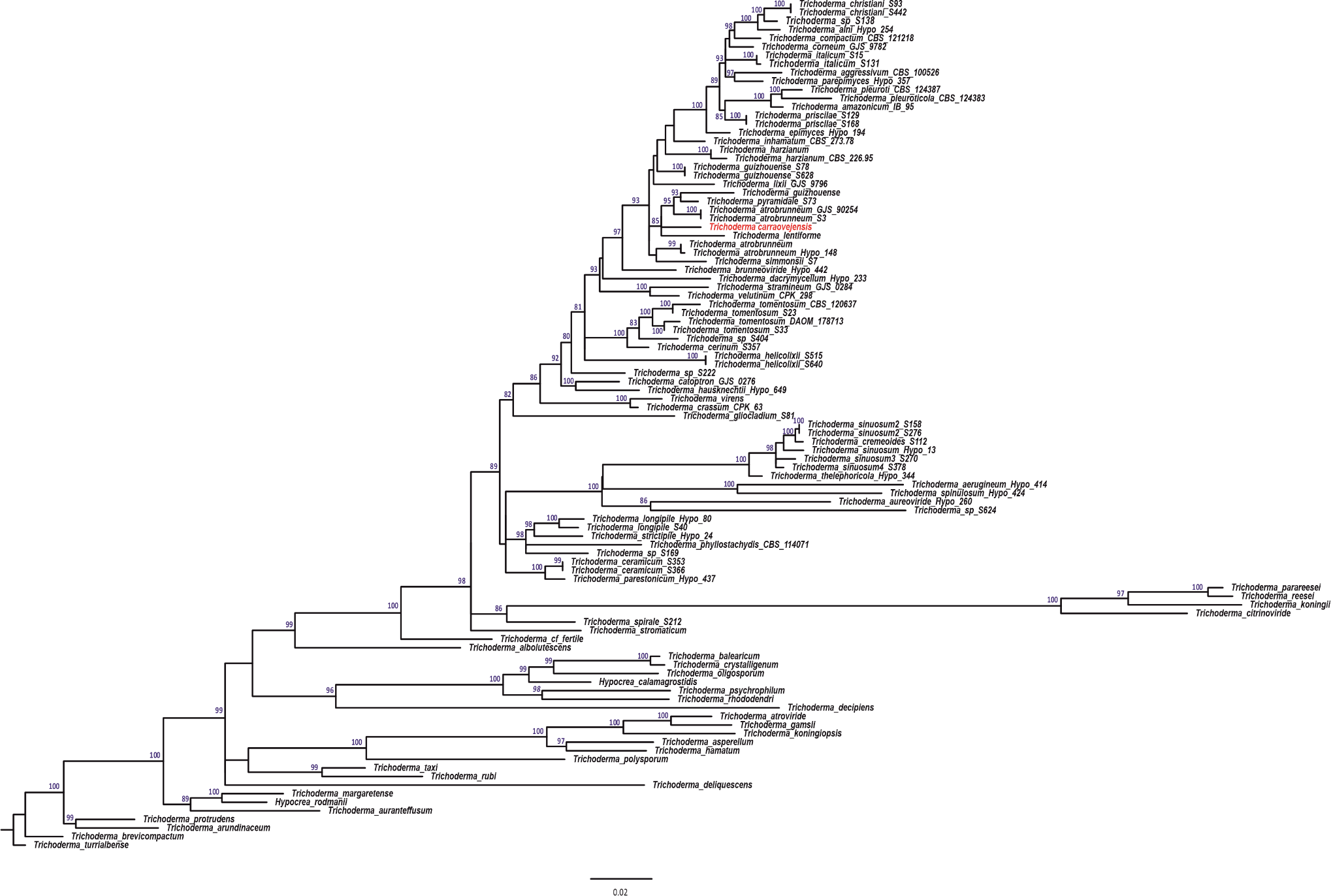


Supplementary Figure S3. Phylogenic tree of the genetic marker *rpb2* (RNA polymerase 2nd largest subunit) using partial amino acid sequences. The sequences were retrieved from different species of Jaklitsch and Voglmayr, 2015.


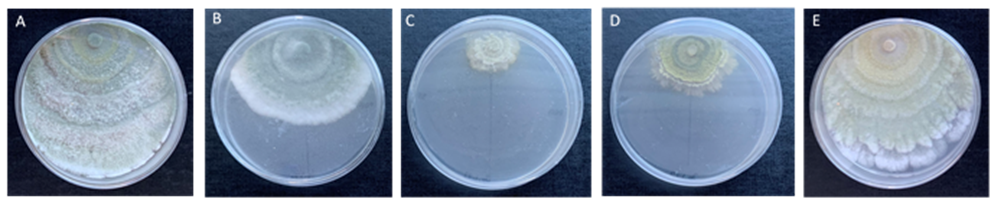

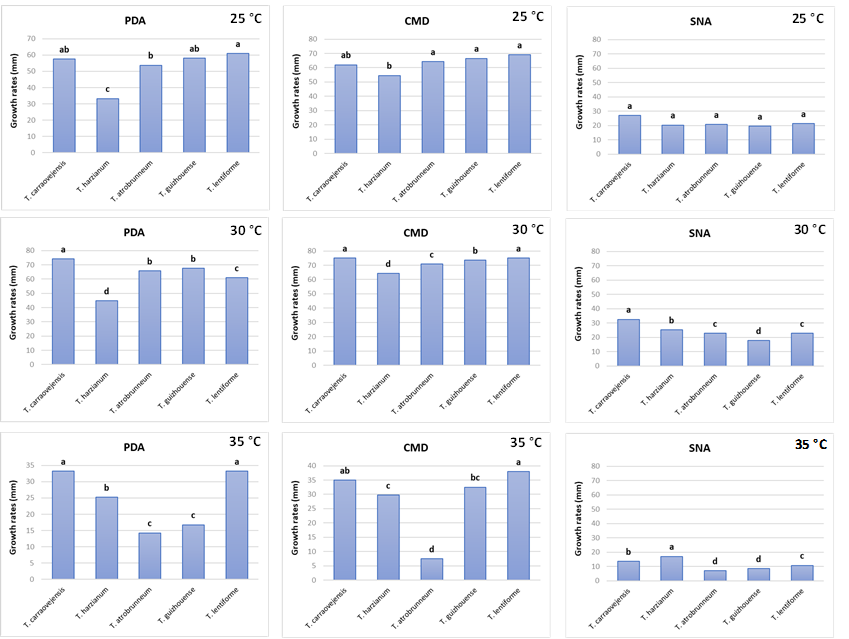


Supplementary Figure S5. Photographs showing the growth in PDA at 35 °C after 7 days. (A) *Trichoderma* *carraovejensis*. (B) *Trichoderma* *harzianum* ULET87. (C) *Trichoderma* *atrobrunneum* CECT 20730. (D) *Trichoderma* *guizhouense* CECT 20731. (E) *Trichoderma* *lentiforme* CBS 100542.

Supplementary Figure S4 . Growth rates in the different culture media at 25, 30 and 35 °C after 72 hours. Different letters indicate significant differences between *Trichoderma* species. Duncan test (p ≤ 0.05).
